# *Lactobacillus plantarum* and *Bifidobacterium longum* Alleviate High-Fat Diet-Induced Obesity and Depression/Cognitive Impairment-like Behavior in Mice by Upregulating AMPK Activation and Downregulating Adipogenesis and Gut Dysbiosis

**DOI:** 10.3390/nu16223810

**Published:** 2024-11-07

**Authors:** Soo-Won Yun, Yoon-Jung Shin, Xiaoyang Ma, Dong-Hyun Kim

**Affiliations:** 1 Neurobiota Research Center, College of Pharmacy, Kyung Hee University, 26, Kyungheedae-ro, Dongdaemun-gu, Seoul 02447, Republic of Korea; ysw6923@naver.com (S.-W.Y.); nayo971111@naver.com (Y.-J.S.); xiaoyangma12@gmail.com (X.M.); 2 PBLbioLab, Inc., Seoul 03174, Republic of Korea

**Keywords:** obesity, liver steatosis, *Lactobacillus plantarum*, *Bifidobacterium longum*, psychiatric disorder, gut microbiota

## Abstract

**Background/Objective:** Long-term intake of a high-fat diet (HFD) leads to obesity and gut dysbiosis. AMP-activated protein kinase (AMPK) is a key regulator of energy metabolism. Herein, we investigated the impacts of *Lactobacillus (Lactiplantibacillus) plantarum* P111 and *Bifidobacterium longum* P121, which suppressed dexamethasone-induced adipogenesis in 3T3 L1 cells and increased lipopolysaccharide-suppressed AMPK activation in HepG2 cells, on HFD-induced obesity, liver steatosis, gut inflammation and dysbiosis, and depression/cognitive impairment (DCi)-like behavior in mice. **Methods:** Obesity is induced in mice by feeding with HFD. Biomarker levels were measured using immunoblotting, enzyme-linked immunosorbent assay, and immunofluorescence staining. **Results:** Orally administered P111, P121, or their mix LpBl decreased HFD-induced body weight gain, epididymal fat pad weight, and triglyceride (TG), total cholesterol (TC), and lipopolysaccharide levels in the blood. Additionally, they downregulated HFD-increased NF-κB activation and TNF-α expression in the liver and colon, while HFD-decreased AMPK activation was upregulated. They also suppressed HFD-induced DCi-like behavior and hippocampal NF-κB activation, NF-κB-positive cell population, and IL-1β and TNF-α levels, while increasing the hippocampal BDNF-positive cell population and BDNF level. The combination of P111 and P122 (LpBl) also improved body weight gain, liver steatosis, and DCi-like behavior. LpBl also mitigated HFD-induced gut dysbiosis: it decreased *Desulfovibrionaceae, Helicobacteriaceae, Coriobacteriaceae,* and *Streptococcaceae* populations and lipopolysaccharide production, which were positively correlated with TNF-α expression; and increased *Akkermansiaceae, Bifidobacteriaceae*, and *Prevotellaceae* populations, which were positively correlated with the BDNF expression. **Conclusions:** P111 and/or P121 downregulated adipogenesis, gut dysbiosis, and NF-κB activation and upregulatde AMPK activation, leading to the alleviation of obesity, liver steatosis, and DCi.

## 1. Introduction

Obesity is the accumulation of abnormal or excessive amounts of fat in the body [1]. The primary risk factor of obesity is the excessive intake of high-calorie diets, such as a high-fat diet (HFD), and physical inactivity [1,2,3]. Long-term HFD feeding causes gut dysbiosis and excessive endotoxin production, which induce inflammation and adipogenesis and suppress the activation of AMP-activated protein kinase (AMPK) [4,5,6]. The AMPK activation downregulates lipid metabolism in cells and organisms, including the oxidative decomposition and biosynthesis of fatty acids and triglycerides [6,7]. Interestingly, endotoxin-induced expression of proinflammatory cytokines suppresses AMPK activation, which inhibits the inflammatory response of adipose tissue macrophages, and systemic inflammation, including neuroinflammation [8,9]. Inflammation-inducing stressors such as pathogen infection and gut bacterial endotoxin induce depression and cognitive impairment (DCi) and systemic inflammation in mice [10,11]. Therefore, suppressing inflammation and adipogenesis and/or inducing AMPK activation may be useful for the therapy of obesity and psychiatric disorders, including DCi.

The gut microbiota is associated with both obesity and psychiatric disorders [5,12]. Gut dysbiosis-ameliorating probiotics may alleviate obesity, depression, and cognitive impairment in mice and volunteers by modulating gut microbiota [13,14,15]. *Lactobacillus delbrueckii* subsp. *bulgaricus strain* TCI904 alleviates HFD-induced weight gain and anxiety in mice [16]. *Lactiplantibacillus* (*Lactobacillus*) *plantarum* LC27 and *Bifidobacterium longum* LC67 alleviate weight gain by suppressing gut bacteria lipopolysaccharide (LPS) production [17]. *Lactobacillus acidophilus* alleviates HFD-induced weight gain, hyperlipidemia, and inflammation in mice [18]. Anti-inflammatory *Lactobacillus plantarum* NK33 and *B. adolescentis* mix (NVP1704) can also alleviate depression/anxiety and systemic inflammation in LPS-producing *Escherichia coli*-exposed mice by modulating the gut microbiota [19]. Nevertheless, studies on obesity-ameliorating action mechanism(s) of probiotics remain elusive.

Therefore, we selected *L. plantarum* P111 and *B. longum* P121, which suppressed dexamethasone-induced fat accumulation in 3T3L1 cells and increased LPS-suppressed AMPK activation in HepG2 cells, from the bacteria collection of healthy human feces and investigated their effects on HFD-induced obesity, liver steatosis, depression, and cognitive impairment in mice.

## 2. Materials and Methods

### 2.1. Materials

LPS (L2630) and DAPI (4,6-diamidine-2-phenylindole dihydrochloride, F6057)) were bought from Sigma (St. Louis, MO, USA). A limulus amoebocyte lysate (LAL) assay kit (113412) was bought from Cape Cod Inc. (E. Falmouth, MA, USA). Antibodies targeting *p*-p65 (#3033), p65 (#6956), AMPK (#25325), and *p*-AMPK (#25315), were bought from Cell Signaling Technology (Danvers, MA, USA). BDNF (PA5-85730) and β-actin (AB8227) were purchased from Santa Cruz Biotechnology (Dallas, TX, USA) and Abcam (Cambridge, UK), respectively. Low-fat diet (LFD, D12450B, Research Diets Inc.) and HFD (D12492) were bought from Research Diets Inc. (New Brunswick, NJ, USA).

### 2.2. Culture of Gut Microbiota-Derived Probiotics and Their Dosage Regimen

Gut bacteria, including P111 (KCCM13475P, from Korean Culture Center of Microorganisms, Seoul, Republic of Korea) and P121 (KCCM13476), were cultured in GAM (D5422, Nissuei Pharm Inc., Tokyo, Japan) or MRS (288130, BD, Franklin Lakes, NJ, USA) broth (0.5 L) and then centrifuged at 5000× *g* for 20 min, washed with saline and distilled water, and freeze-dried. The freeze-dried cells were resuspended in phosphate-buffered saline for in vitro cell experiment or 1% trehalose for in vivo animal experiment.

To determine the appropriate dose of probiotics for in vivo studies, mice were subjected to oral gavage with P111 (2 × 10^8^ and 1 × 10^9^ colony-forming unit (CFU)/mouse/day) for 4 weeks in conjunction with HFD. Compared to mice receiving only HFD treatment, P111 at doses of 2 × 10^8^ CFU/mouse and 1 × 10^9^ CFU/mouse reduced weight gain by 22% and 36%, respectively. Consequently, a dose of 1 × 10^9^ CFU/mouse/day was selected for further in vivo experiments.

### 2.3. HepG2 and 3T3-L1 Cell Cultures

HepG2 cells (Korean Cell Line Bank, Seoul, Republic of Korea) were cultured in DMEM (00741, GIBCO, Grand Island, NY, USA) containing 1% antibiotic–antimycotic (AA, 15240-062, GIBCO) and 10% fetal bovine serum (FBS, 26140-079, GIBCO) at 37 °C in a 5% CO_2_/air atmosphere [20]. The cells (1 × 10^6^ cells/mL) were treated with or without probiotics (1 × 10^5^ CFU/mL) in the presence of palmitic acid (0.25 mM) or LPS (100 ng/mL) for 24 h. 3T3-L1 cells (American Type Culture Collection, Manassas, VA, USA) were cultured in DMEM supplemented with 10% FBS and 1% AA at 37 °C and 5% CO_2_/air and differentiated into the adipocyte, as previously reported [21].

Lipid amount and AMPK activation activity were assayed using Oil Red O staining and immunoblotting, respectively, as previously reported [21].

### 2.4. Animals

Animal experiments were conducted using male C57BL/6 mice (18–21 g, 6 weeks old) obtained from Koatech (Pyeongtaek, Republic of Korea). Mice were housed in plastic cages with 5 cm elevated wire flooring, under controlled conditions, for one week prior to the commencement of experiments and used in animal experiments, as previously reported [19]. All animal experiments were approved by the Institutional Animal Care and Use Committee (IACUC approval nos. KHSASP-20-177, 14 July 2020; and KHSASP-21-098, 11 March 2021) and conducted according to the Ethical Policies and Guidelines of the University for Laboratory Animals Care and Use and Use of Laboratory Animals and ARRIVE guideline [22].

### 2.5. Preparation of Mice with Obesity and Anti-Obesity Activity Assay of Probiotics

To understand the anti-obesity activities of Lpl, Blo, and their (4:1) mix (LpBl), we examined two sequential experiments in mice with HFD-induced obesity, which were prepared as previously reported [17]. First, to investigate the effects of Lpl and Blo, mice were randomly separated into 4 groups (LF, HF, Lpl, and Blo). Second, to examine the effects of LpBl, mice were randomly separated into 3 groups (LF, HF, and LpBl). Each group consisted of 8 mice. The LF group was fed an LFD for 8 weeks, while the HF, Lpl, Blo, and LpBl groups were subjected to an HFD for the same duration. The LF and HF groups were administered 1% trehalose (vehicle) via oral gavage once a day (six day/week) after feeding on their assigned diet for 4 weeks. Lpl, Blo, and LpBl groups were administered P111, P121, and their (4:1) mix (1 × 10^9^ CFU/mouse/day, suspended in 1% trehalose) via oral gavage once a day (six day/week) from next day after HFD feeding for 4 weeks, respectively.

DCi-like behaviors were measured 24 h after the final gavage of probiotics. Mice were euthanized via exposure to CO_2_, followed by cervical dislocation. Blood, colon, liver, and brain tissues were collected and stored at −80 °C for biochemical marker analysis.

### 2.6. Behavioral Tasks

Depression-like behaviors were assessed using the elevated plus-maze test (EPMT) and tail suspension test (TST) performed in a plus-maze apparatus and at the edge of a table, respectively, as previously described [19]. Cognitive function-like behaviors were evaluated using the Y-maze task (YMT) conducted in a three-arm horizontal maze (40 cm long, 3 cm wide, and 12 cm high walls), as previously reported [23]. Detailed protocols are indicated in the Supplementary Materials’ Methods section.

### 2.7. Immunoblotting and ELISA

Colon, liver, and brain tissue samples were homogenized and lysed in RIPA buffer and then centrifuged at 14,000× *g* for 20 min. Proteins in the supernatant (20 μg) were analyzed by immunoblotting for p65, *p*-p65, AMPK, *p*-AMPK, BDNF, and β-actin, as previously described [23]. Cytokines in the liver, colon, and blood supernatants were measured using ELISA kits [17].

### 2.8. Determination of LPS Concentration

LPS levels in blood, liver, and feces were measured using an LAL assay kit, as previously reported [17].

### 2.9. Determination of Total Cholesterol (TC), HDL-Cholesterol (HC), and Triglyceride (TG) Levels in the Liver and Blood

Liver tissue samples were homogenized, lysed in RIPA buffer (pc2002-050-00, Biosesang, Yongin-si, Republic of Korea), and then centrifuged at 14,000 g for 20 min. TC, HC, and TG levels in the liver homogenate supernatant and blood were measured using each commercial kit (Asan pharmaceutical Co., Seoul, Republic of Korea).

### 2.10. Immunofluorescence Staining

Immunofluorescence staining was performed, as described by Jang et al. [19]. Detailed protocols are provided in the Supplementary Materials (Methods).

### 2.11. Quantitative Real-Time Polymerase Chain Reaction (qPCR)

Quantitative PCR (qPCR) was performed for (SIRT)-1, sREBP-1c, PGC-1α, LPL, Fiaf, G6PD, FAS, and β-actin, following the method described by Jang et al. [19]. The primers are indicated in Supplementary Table S1.

### 2.12. Gut Microbiota Composition Analysis

Gut microbiota composition was determined using Illumina iSeq 100 [23]. Detailed protocols are provided in the Supplementary Materials (Methods). Sequenced reads were deposited in the NCBI’s short-read archive under accession number PRJNA163520.

### 2.13. Whole-Genome Analysis

The whole-genome sequences of P111 and P121 were analyzed, as previously reported [23]. Detailed protocols are provided in the Supplementary Materials (Methods).

### 2.14. Statistical Analysis

Data were indicated as the mean ± standard deviation (SD) and analyzed by a GraphPad Prism 9. The significance was analyzed by a one-way ANOVA, followed by Duncan’s multiple-range test (*p* < 0.05).

## 3. Results

### 3.1. Effects of Probiotics on Lipid Accumulation and AMPK Activation in 3T3-L1 and HepG2 Cells

**Figure 1:**
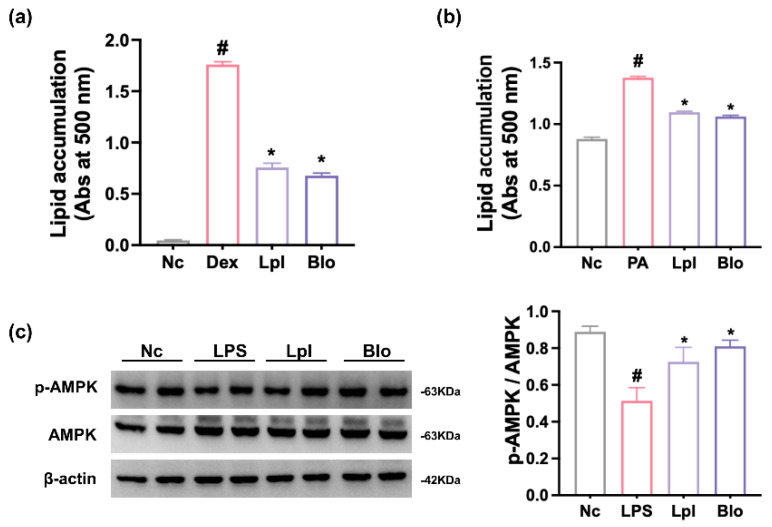
Effects of P111 and P121 on fat (lipid) deposition and AMPK activation in 3T3 L1 and HepG2 cells. (**a**) Effects on fat deposition in 3T3 L1 cells. Nc, vehicle alone; Dex, dexamethasone; Lpl, P111+dexamethasone; Blo, P121+dexamethasone. (**b**) Effects on fat deposition in HepG2 cells. Nc, vehicle alone; PA, palmitic acid; Lpl, P111+palmitic acid; Blo, P121+palmitic acid. (**c**) Effects on AMPK activation in HepG2 cells. Nc, vehicle alone; LPS, lipopolysaccharide (LPS); Lpl, P111+LPS; Blo, P121+LPS. Fat deposition and AMPK activation were assessed by Oil Red O staining and immunoblotting, respectively. 3T3 L1 cells were treated with probiotics (1 × 10^5^ CFU/mL) and dexamethasone. HepG2 cells were treated with probiotics (1 × 10^5^ CFU/mL) and palmitic acid. (n = 4). ^#^ *p* < 0.05 vs. NC. * *p* < 0.05 vs. Dex or PA alone.

First, we screened probiotics suppressing lipid accumulation in 3T3 L1 cells from healthy human fecal microbiota-derived lactic acid bacteria collection. Of them, P111 and P121 significantly suppressed dexamethasone-mediated adipogenesis (fat accumulation) in 3T3 L1 cells (Figure 1 and Supplementary Figure S1). They also suppressed palmitic acid-induced fat (lipid) accumulation in HepG2 cells. When they were (4:1), (1:1), or (1:4) mixed, their lipid accumulation-inhibitory effects were not different. However, they increased LPS-suppressed AMPK activation in HepG2 cells. Based on the analysis of Gram staining, whole genome and 16S rRNA gene, and API 20A and 50 CHL kits (bioMérieux, Marcy-l’Étoile, France), P111 and P121 were named *L. plantarum* and *B. longum*, respectively. Their whole-genome sequences exhibited the highest phylogenetic similarity to *L. plantarum* NCTC13644 (99.1%) and *B. longum* DSM20211 (96.3%), respectively, using OrthoANI (Supplementary Figure S2).

### 3.2. P111 and P121 Alleviated HFD-Induced Body Weight Gain, Liver Steatosis, and Depression in Mice

**Figure 2:**
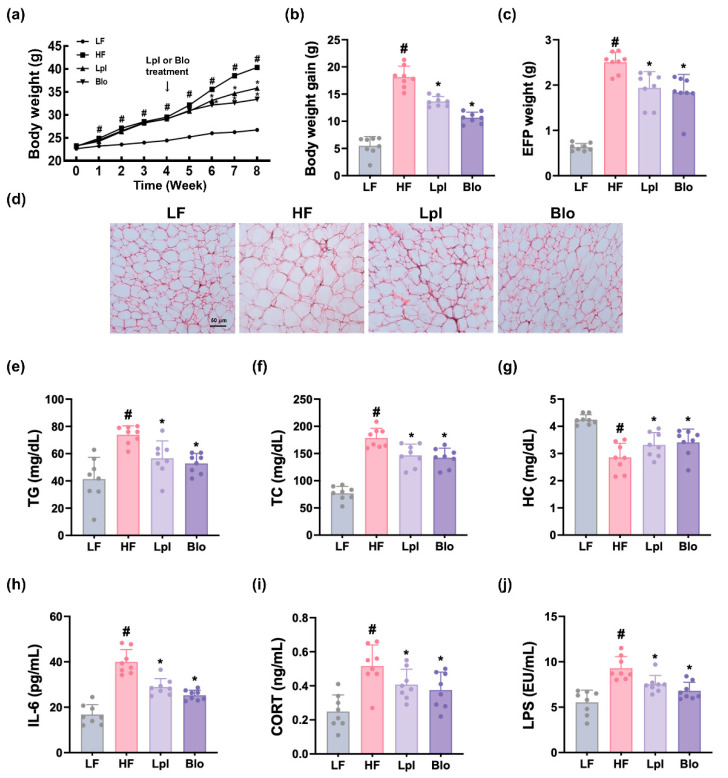
Effects of P111 and P121 on HFD-increased obesity in mice. Effects on body weight change (**a**), body weight gain (**b**), EFP weight (**c**), and EFP adipocyte size (**d**). Effects on TG (**e**), TC (**f**), and HC levels (**g**) in the blood. Effects on IL-6 (**h**), corticosterone (CORT, (**i**)), and LPS levels (**j**) in the blood. LF, LFD (8 weeks) alone; HF, HFD (8 weeks) alone; Lpl, P111 (4 weeks) with HFD (8 weeks); Blo, P121 (4 weeks) with HFD (8 weeks). n = 8. ^#^ *p* < 0.05 vs. LF group. * *p* < 0.05 vs. HF group.

The effects of P111 and P121 on body weight changes were investigated in mice subjected to HFD. Long-term feeding of HFD significantly increased body weight gain compared to those of LFD feeding (Figure 2). Oral administration of P111 or P121 effectively mitigated HFD-induced weight gain. They also increased HFD-induced epididymal fat pad (EFP) weight and adipocyte size, as assessed by H&E staining.

HFD feeding also increased TG, TC, and HC levels in the blood. However, oral administration of P111 or P121 reduced HFD-increased TG and TC, while the HFD-decreased HC level increased. They also decreased HFD-induced corticosterone, IL-6, and LPS levels in the blood.

**Figure 3:**
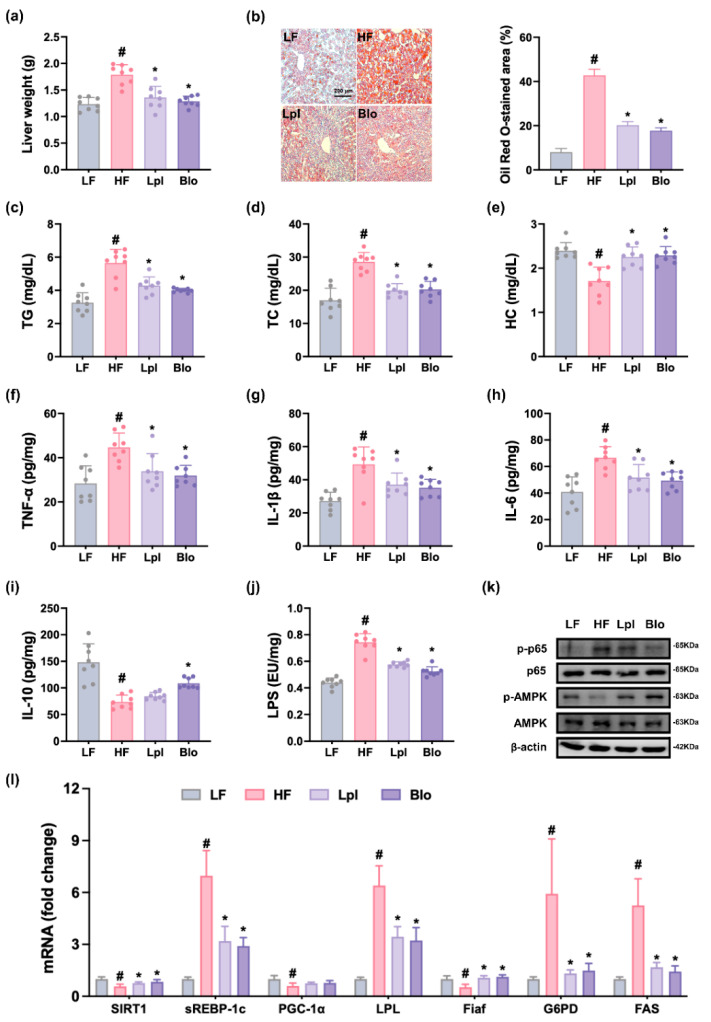
Effects of P111 and P121 on liver weight and steatohepatitis-related marker expression in the liver. Effects on liver weight (**a**) and lipid droplet number (**b**). Effects on TG (**c**), TC (**d**), and HC (**e**). Effects on TNF-α (**f**), IL-1β (**g**), IL-6 (**h**), IL-10 (**i**), and LPS (**j**) levels, as assessed by ELISA. (**k**) Effects on *p*-p65, p65, *p*-AMPK, AMPK, and β-actin expression, as assessed by immunoblotting. (**l**) Effects on SIRT1, sREBP-1c, PGC-1a, LPL, Fiaf, G6PD, and FAS levels, as assessed by qPCR. LF, LFD (8 weeks) alone; HF, HFD (8 weeks) alone; Lpl, P111 (4 weeks) with HFD (8 weeks); Blo, P121 (4 weeks) with HFD (8 weeks). n = 8. ^#^ *p* < 0.05 vs. LF group. * *p* < 0.05 vs. HF group.

HFD feeding increased liver weight and lipid droplet number (Figure 3 and Supplementary Figure S3). Treatment with HFD also increased TG, TC, and HC levels in the liver. However, P111 and P121 significantly reduced HFD-induced liver weight; lipid droplet number; and TG, TC, and HC levels. Additionally, their treatments reduced TNF-α, IL-1β, IL-6, and LPS levels.

HFD feeding suppressed AMPK activation and induced NF-κB activation in the liver. Furthermore, HFD feeding decreased SIRT-1, PGC-1α, and Fiaf levels, while sREBP-1c, LPL, G6PD, and FAS levels increased. However, treatment with P111 or P121 induced HFD-suppressed AMPK activation and SIRT-1, PGC-1α, and Fiaf levels, while HFD-induced sREBP-1c, LPL, G6PD, and FAS levels and NF-κB activation decreased.

**Figure 4:**
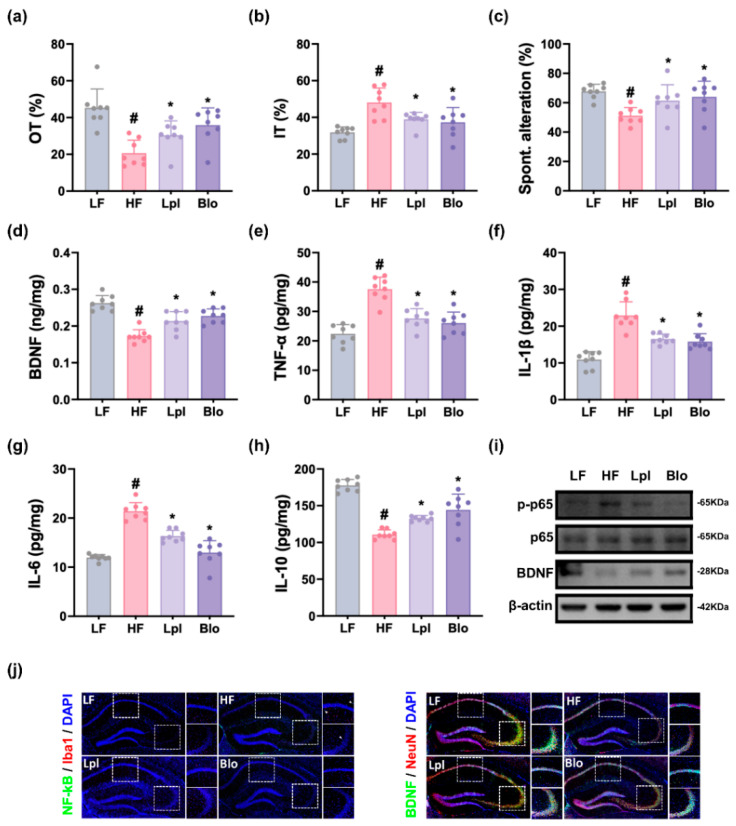
Effects of P111 and P121 on DCi-like symptoms in mice with HFD-induced obesity. Effects on OT in the EPMT (**a**), IT in the TST (**b**), and SA in the YMT (**c**). Effects on hippocampal BDNF (**d**), TNF-α (**e**), IL-1β (**f**), IL-6 (**g**), and IL-10 expression (**h**). (**i**) Effects on hippocampal *p*-p65, p65, BDNF, and β-actin expression. (**j**) Effects on hippocampal NF-κB^+^Iba1^+^ and BDNF^+^NeuN^+^ cell numbers. LF, LFD (8 weeks) alone; HF, HFD (8 weeks) alone; Lpl, P111 (4 weeks) with HFD (8 weeks); Blo, P121 (4 weeks) with HFD (8 weeks). n = 8. ^#^ *p* < 0.05 vs. LF group. * *p* < 0.05 vs. HF group.

Long-term feeding of HFD increased depression-like behaviors, time spent in open arm (OT) and open arm entries (OE) in the EPMT, to 45.6% (F_3,28_ = 11.12, *p* < 0.001) and 50.0% (F_3,28_ = 4.56, *p* < 0.01) of LFD-fed mice, respectively, and immobility time (IT) in the TST to 151.3% (F_3,28_ = 9.73, *p* < 0.001) of LFD-fed mice, respectively (Figure 4 and Supplementary Figure S4). HFD also decreased spontaneous alternation (SA) in the YMT to 75.5% (F_3,28_ = 5.71, *p* < 0.001) of LFD-fed mice. Orally treated P111 and P121 alleviated HFD-induced depression-like behaviors OT to 67.2% and 67.9% of LFD-fed mice, respectively, and IT to 122.4% and 124.8% of LFD-fed mice, respectively. They recovered HFD-decreased SA to 90.6% and 88.6% of LFD-fed mice, respectively.

HFD treatment increased TNF-α, IL-1β, and IL-6 levels; NF-κB activation; and NF-κB^+^Iba1^+^ cell number in the hippocampus, while decreasing BDNF levels and the BDNF^+^NeuN^+^ cell number. However, oral treatment with P111 or P121 reduced HFD-induced TNF-α, IL-1β, and IL-6 levels; NF-κB activation; and NF-κB^+^ cell number, while increasing the BDNF level and BDNF^+^NeuN^+^ cell number suppressed by HFD.

### 3.3. P11 and P121 Alleviated HFD-Induced Gut Inflammation and Dysbiosis in Mice

**Figure 5:**
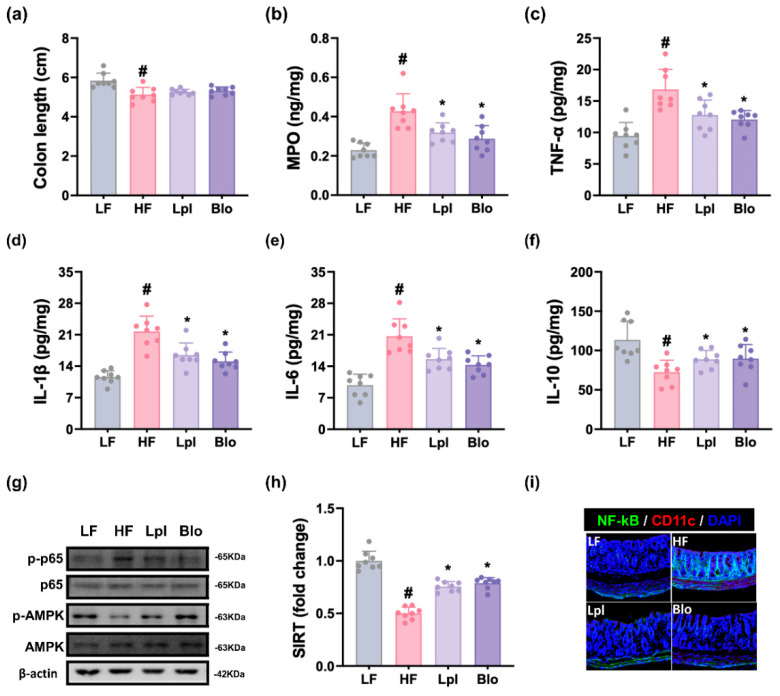
Effects of P111 and P121 on HFD-induced colitis in mice. (**a**) Effects on colon length. Effects on colonic myeloperoxidase (MPO, (**b**)), TNF-α (**c**), IL-1β (**d**), IL-6 (**e**), and IL-10 expression (**f**), as assessed by ELISA. (**g**) Effects on colonic *p*-p65, p65, *p*-AMPK, AMPK, and β-actin expression, as assessed by immunoblotting. (**h**) Effects on colonic SIRT1 expression, as assessed by qPCR. (**i**) Effects on colonic NF-κB^+^CD11c^+^ cell populations. LF, LFD (8 weeks) alone; HF, HFD (8 weeks) alone; Lpl, P111 (4 weeks) with HFD (8 weeks); Blo, P121 (4 weeks) with HFD (8 weeks). n = 8. ^#^ *p* < 0.05 vs. LF group. * *p* < 0.05 vs. HF group.

Long-term HFD feeding shortened the length of colon and enhanced the expression of myeloperoxidase, TNF-α, IL-1β, and IL-6, activation of NF-κB activation (*p*-p65/p65), and number of NF-κB^+^CD11c^+^ cells in the colon, while decreasing IL-10 and SIRT1 expression and AMPK activation (*p*-AMPK/AMPK) (Figure 5 and Supplementary Figure S5). Orally administered P111 or P121 suppressed HFD-induced myeloperoxidase; IL-1β, IL-6, and TNF-α levels; NF-κB activation (*p*-p65/p65); and NF-κB-positive cell population and enhanced HFD-decreased IL-10 expression and AMPK activation (*p*-AMPK/AMPK).

### 3.4. Effects of P111 and P121 Mix (LpBl) on HFD-Induced Body Weight, Liver Steatosis, and Their Related Biomarker Levels in Mice

**Figure 6:**
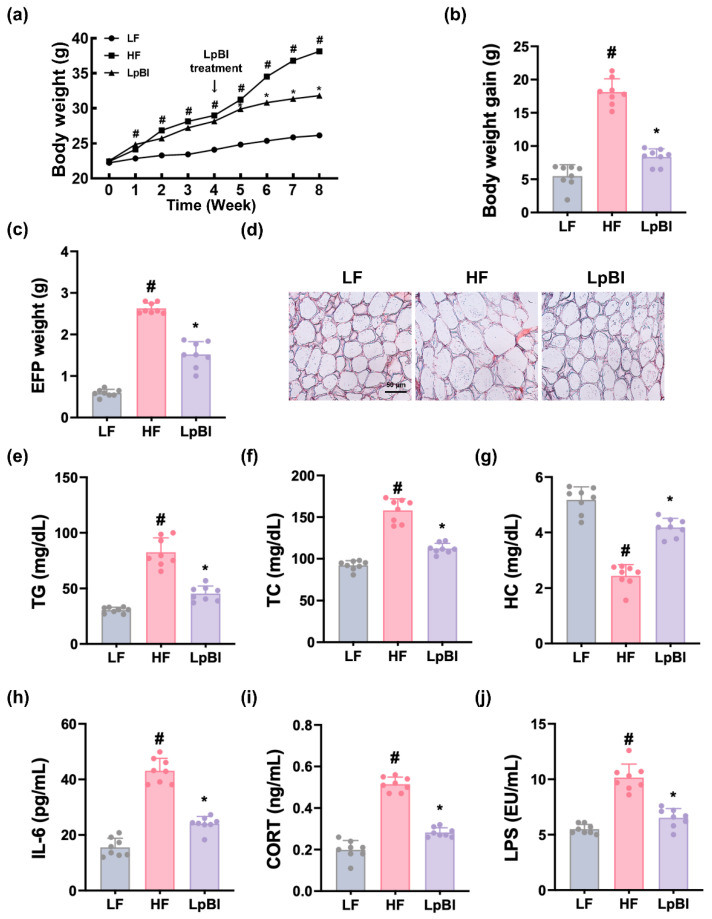
Effect of LpBl on HFD-induced body weight, liver steatosis, and their related biomarker levels in mice. Effect on body weight change (**a**), body weight gain (**b**), EFP weight (**c**), and EFP adipocyte size (**d**). Effects on blood TG (**e**), TC (**f**), and HC levels (**g**). Effect on blood IL-6 (**h**), corticosterone (CORT, (**i**)), and LPS levels (**j**), as assessed by ELISA. LF, LFD (8 weeks) alone; HF, HFD (8 weeks) alone; LpBl, P111 and P121 mix (4 weeks) with HFD (8 weeks). n = 8. ^#^ *p* < 0.05 vs. LF group. * *p* < 0.05 vs. HF group.

When they were (4:1), (1:1), or (1:4) mixed, the lipid accumulation-inhibitory effects were not different in palmitic acid-treated HepG2 cells. Therefore, we examined the effect of LpBl (P111 and P121 (4:1) mix) on HFD-increased body weight gain in mice (Figure 6). HFD feeding significantly increased body weight gain compared to LFD feeding. However, oral administration of LpBl decreased HFD-induced body weight gain. Furthermore, they also decreased HFD-induced EFP weight and adipocyte size.

HFD feeding enhanced blood TG, TC, and HC levels. On the contrary, oral treatment with LpBl decreased HFD-induced TG and TC, while the HFD-suppressed HC level increased. LpBl also reduced HFD-induced blood IL-6, corticosterone, and LPS levels.

**Figure 7:**
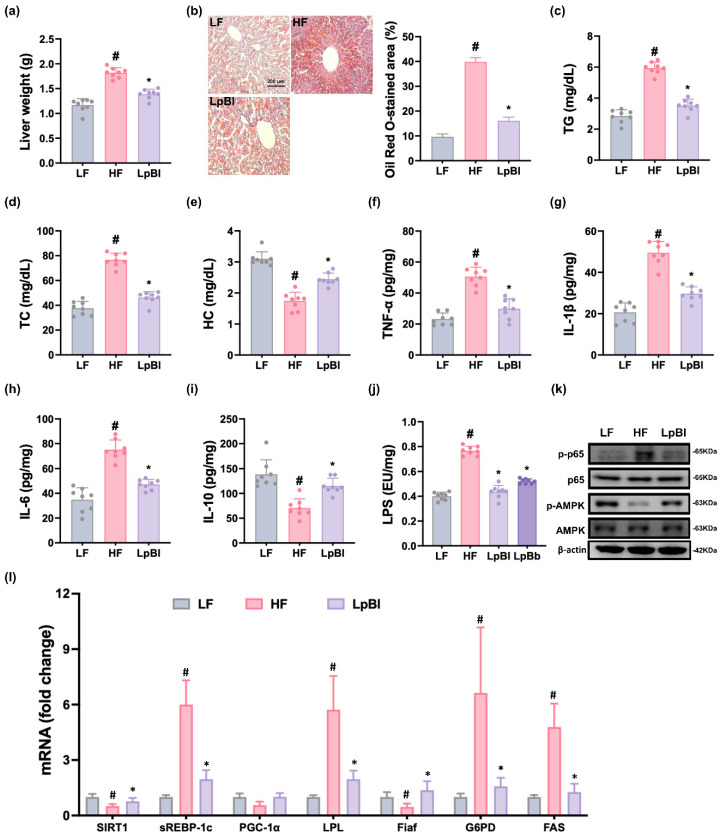
Effect of LpBl (P111 and P121 (4:1) mix) on liver steatosis-related marker levels. Effects on liver weight (**a**) and lipid droplet number (**b**). Effects on liver TG (**c**), TC (**d**), and HC (**e**). Effects on liver TNF-α (**f**), IL-1β (**g**), IL-6 (**h**), IL-10 (**i**), and LPS levels (**j**), as assessed by ELISA. (**k**) Effects on liver *p*-p65, p65. P-AMPK, AMPK, and β-actin levels, as assessed by immunoblotting. (**l**) Effects on liver SIRT1, sREBP-1c, PGC-1a, LPL, Fiaf, G6PD, and FAS levels, as assessed by qPCR. LF, LFD (8 weeks) alone; HF, HFD (8 weeks) alone; LpBl, P111 and P121 mix (4 weeks) with HFD (8 weeks). n = 8. ^#^ *p* < 0.05 vs. LF group. * *p* < 0.05 vs. HF group.

HFD feeding also increased liver weight, lipid droplet number, and elevated levels of TG, TC, and HC in the liver (Figure 7 and Supplementary Figure S6). Conversely, oral treatment with LpBl significantly lowered HFD-increased liver weight; lipid droplet number; and TG, TC, and HC levels. Additionally, LpBl treatment attenuated HFD-induced elevations in TNF-α, IL-1β, IL-6, and LPS levels and NF-κB activation.

Furthermore, LpBl upregulated HFD-decreased AMPK activation and PGC-1α, SIRT-1, and Fiaf levels, while HFD-induced sREBP-1c, LPL, G6PD, and FAS expression levels decreased.

**Figure 8:**
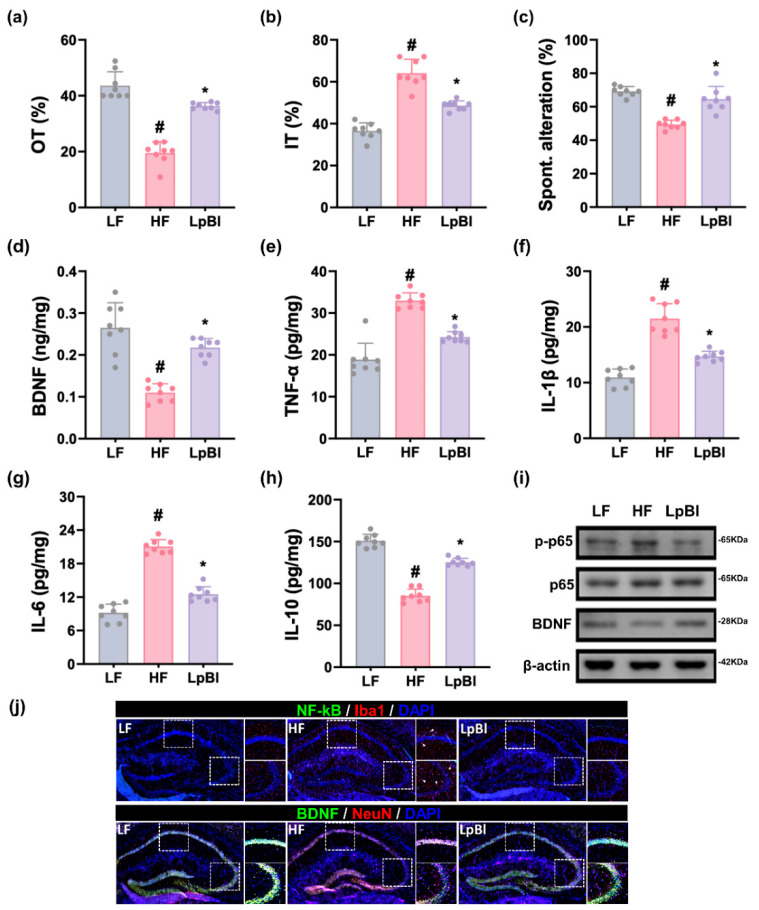
Effect of LpBl on HFD-induced DCi in mice. Effects on OT (**a**) in EPMT, IT in TST (**b**), and SA in YMT (**c**). Effect on hippocampal BDNF (**d**), TNF-α (**e**), IL-1β (**f**), IL-6 (**g**), and IL-10 levels (**h**), as assessed by ELISA. (**i**) Effect on hippocampal *p*-p65, p65, BDNF, and β-actin levels, as assessed by immunoblotting. (**j**) Effect on hippocampal NF-κB^+^Iba1^+^ and BDNF^+^NeuN^+^ cell number, as assessed by the confocal microscope. LF, LFD (8 weeks) alone; HF, HFD (8 weeks) alone; LpBl, P111 and P121 mix (4 weeks) with HFD (8 weeks). n = 8. ^#^ *p* < 0.05 vs. LF group. * *p* < 0.05 vs. HF group.

HFD feeding decreased OT and OE in the EPMT to 44.7% (F_2,21_ = 87.09, *p* < 0.001) and 34.7% (F_2,21_ = 104.2, *p* < 0.001) of LFD-fed mice, respectively, and increased IT in the TST to 175.4% (F_2,21_ = 71.99, *p* < 0.001) of LFD-fed mice, respectively (Figure 8 and Supplementary Figure S7). HFD treatment also decreased SA in the YMT to 71.5% (F_2,21_ = 35.61, *p* < 0.001) of LFD-fed mice. Conversely, oral treatment with LpBl alleviated HFD-induced DCi-like behaviors: it recovered OT to 83.2% of LFD-fed mice, IT to 133.0% of LFD-fed mice, and SA to 93.4% of LFD-fed mice.

Furthermore, LpBl treatment significantly lowered HFD-induced levels of TNF-α, IL-1β, and IL-6; activation of NF-κB; and number of NF-κB-positive cells in the hippocampus. Conversely, LpBl treatment enhanced HFD-suppressed levels of BDNF and the number of BDNF-positive cells.

### 3.5. Effect of LpBl on HFD-Induced Gut Inflammation and Dysbiosis in Mice

**Figure 9:**
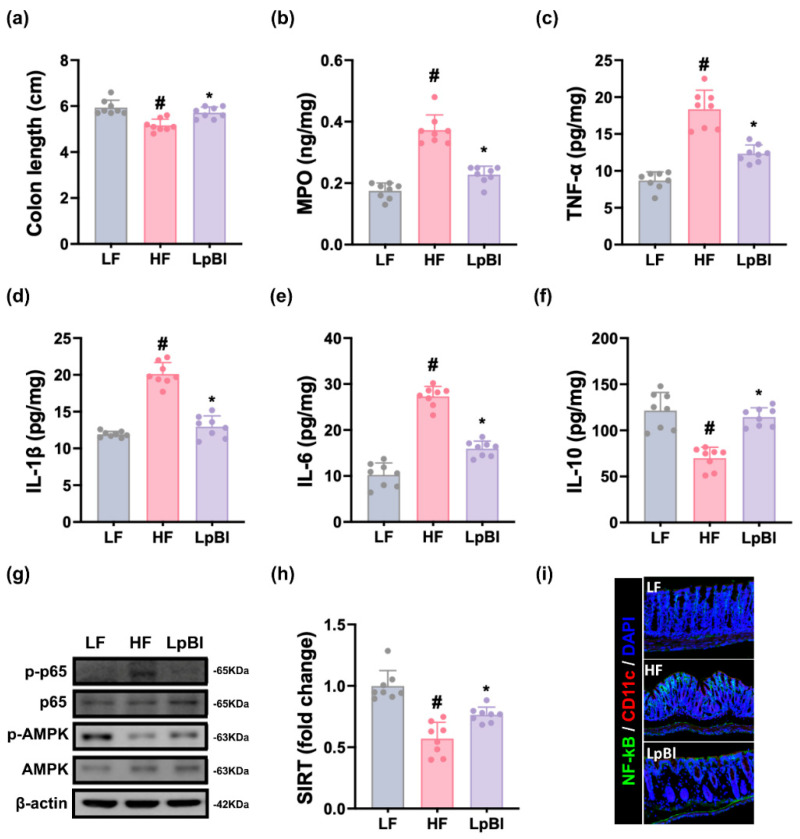
Effect of LpBl on HFD-induced gut inflammation in mice. (**a**) Effect on colon length. Effect on colonic myeloperoxidase (MPO, (**b**)), TNF-α (**c**), IL-1β (**d**), IL-6 (**e**), and IL-10 levels (**f**), as assessed by ELISA. (**g**) Effect on colonic *p*-p65, p65, *p*-AMPK, AMPK, and β-actin levels, as assessed by immunoblotting. (**h**) Effect on colonic SIRT1 level, as assessed by qPCR. (**i**) Effect on colonic NF-κB^+^CD11c^+^ cell number. LF, LFD (8 weeks) alone; HF, HFD (8 weeks) alone; LpBl, P111, and P121 mix (4 weeks) with HFD (8 weeks). n = 8. ^#^ *p* < 0.05 vs. LF group. * *p* < 0.05 vs. HF group.

HFD feeding resulted in colitis: it elevated myeloperoxidase, TNF-α, IL-1β, and IL-6 levels, NF-κB activation (*p*-p65/p65), and NF-κB^+^CD11c^+^ cell number and decreased IL-10 and SIRT1 levels and AMPK activation (*p*-AMPK/AMPK) in the colon (Figure 9 and Supplementary Figure S8). However, LpBl treatment lowered HFD-induced myeloperoxidase; IL-1β, IL-6, and TNF-α levels; NF-κB activation (*p*-p65/p65); and NF-κB-positive cell population and enhanced HFD-suppressed IL-10 level and AMPK activation (*p*-AMPK/AMPK).

**Figure 10:**
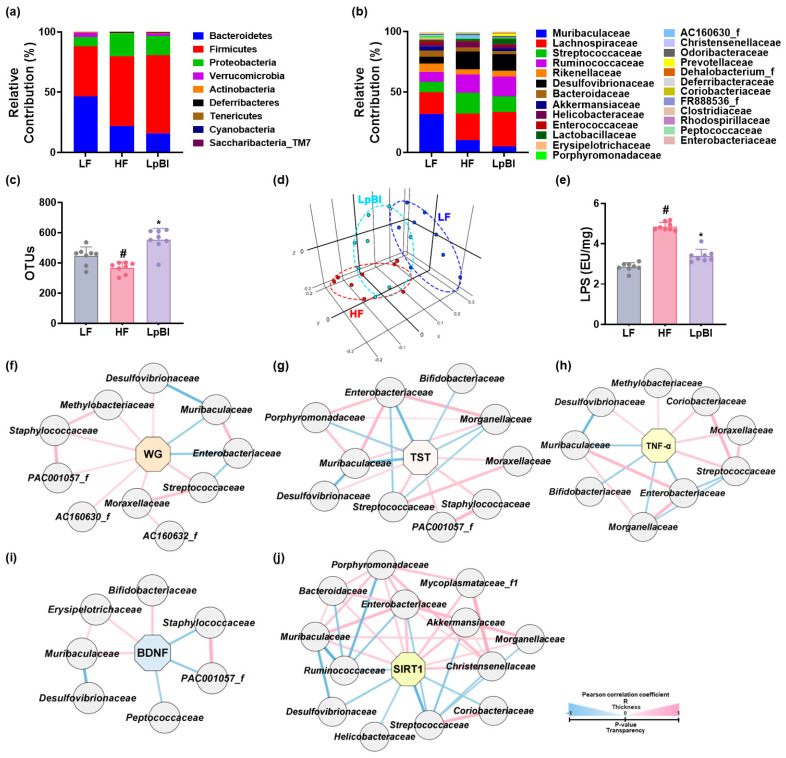
Effect of LpBl on HFD-induced gut dysbiosis in mice. Effect on the composition of fecal microbiota: (**a**) phylum and (**b**) family levels; (**c**) OTU (α-diversity); and (**d**) β-diversity (PCoA plot). (**e**) Effects on fecal microbiota LPS level. Network analysis between gut microbiota and body weight gain (WG, (**f**)), IT in the TST (**g**), TNF-α (**h**), BDNF (**i**), or SIRT1 level (**j**). LF, LFD (8 weeks) alone; HF, HFD (8 weeks) alone; LpBl, P111, and P121 mix (4 weeks) with HFD (8 weeks). n = 8. ^#^ *p* < 0.05 vs. LF group. * *p* < 0.05 vs. HF group.

We examined the impact of LpBl on HFD-induced gut dysbiosis in mice. HFD feeding also fluctuated the composition of gut microbiota: it decreased α-diversity (OUT richness) and shifted β-diversity compared to that of LFD-fed mice (Figure 10 and Supplementary Tables S2 and S3). However, oral administration of LpBl increased HFD-suppressed α-diversity and partially shifted HFD-changed β-diversity to that of LFD-fed mice. In particular, HFD feeding increased the populations of *Firmicutes* and *Proteobacteria*, including *Streptococcaceae*, *Ruminococcaceae*, *Desulfovibrionaceae*, *Helicobacteriaceae*, *Coriobacteriaceae*, *AC160630_f*, and *Peptococcaceae*, while the populations of *Bacteroidetes* and *Verrucomicrobia*, including *Muribaculaceae*, *Akkermansiaceae*, *Prevotellaceae*, *Porphyromonadaceae*, *FR888536_f*, and *Bifidobacteriaceae*, decreased. However, oral administration of LpBl partially shifted the HFD-changed gut microbiota composition to that of LFD-fed mice. In particular, LpBl decreased *Streptococcaceae*, *Helicobacteriacae*, *Peptococcaceae*, and *AC160630_f* in mice with HFD-induced obesity, while *Akkermansiaceae*, *Prevotellaceae*, *Lactobacillaceae*, and *Bifidobacteriaceae* populations increased. LpBl also decreased HFD-induced fecal LPS levels.

At a family level, *Streptococcaceae*, *Desulfovibrionaceae*, and *Moraxellaceae* populations had a positive correlation with body weight gain, while *Muribacullaceae* and *Enterobacteriaceae* populations were negatively correlated. *Staphylococcaceae*, *Moracellaceae*, *Streptococcaceae*, and *Desulfovibrionaceae* populations had a positive correlation with depression-like behaviors (IT in TST), while *Muribaculaceae*, *Enterobacteriaceae*, *Bifidobacteriaceae*, *Bifidobacteriaceae*, and *Morganellaceae* populations were negatively correlated. Liver TNF-α expression level had a positive correlation with *Moraxellaceae*, *Streptococcaceae*, *Corobacteriaceae*, and *Desulfovibrionaceae* populations, while *Enterobacteriacae*, *Bifidobacteriaceae*, *Muribaculaceae*, and *Morganellaceae* populations were negatively correlated. Hippocampal BDNF expression levels had a positive correlation with *Bifidobacteriaceae*, *Muribaculaceae*, and *Erysipelotrichaceae* populations, while *Peptococcaceae*, *Staphylococcaceae*, and *PAC001057_f* populations were negatively correlated. Liver SIRT1 expression level had a positive correlation with *Akkermansiaceae*, *Porphyromonadaceae*, *Enterobacteriaceae*, *Muribaculaceae*, and *Bacteroidaceae* populations, while *Streptococcaceae*, *Desulfovibrionaceae*, *Helicobacteriaceae*, and *Corobacteriaceae* populations were negatively correlated.

## 4. Discussion

Excessive, chronic feeding of an HFD induces obesity, which is the representative risk factor for heart disease, diabetes mellitus, and hepatic steatosis [24,25]. Furthermore, HFD induces gut dysbiosis and microbiota LPS production in humans and mice, inducing gut inflammation and membrane permeability [6,26]. Excessively exposed LPS also suppresses AMPK activation and induces adipogenesis in the gut and liver and causes neuroinflammation [7,27]. We also found that chronic feeding of an HFD induced body weight gain; inflammation in the colon, liver, and hippocampus; and DCi-like behavior in mice.

In the present study, oral administration of P111, P121, or LpBL decreased HFD-induced body weight gain and EFP weight. Furthermore, they enhanced HFD-suppressed AMPK activation and SIRT expression in both the colon and liver. They also increased HFD-suppressed PGC-1α and Fiaf expression and decreased HFD-induced sREBP-1c, LPL, G6PD, and FAS expression. The feeding of an HFD decreases AMPK activation, which regulates the expression of lipogenesis/lipolysis-involved metabolism-regulatory factors PGC1α, sREBP-1c, Fiaf, LDL, G6PD, and SIRT-1 [28,29,30,31]. An HFD suppresses SIRT1 expression, which increases PGC-1α expression and AMPK activation [28,32]. P111, P121, and LpBL suppressed lipid accumulation and induced AMPK activation in palmitic acid-stimulated HepG2 cells and suppressed adipogenesis in dexamethasone-stimulated 3T3 L1 cells. These findings imply that P111, P121, or LpBL are able to suppress lipogenesis and induce lipolysis in the liver and intestine by inducing AMPK activation and SIRT1 expression, leading to the alleviation of liver steatosis. They also decreased HFD-induced liver and EFP weights; liver lipid droplet number; and TC, TG, and HC levels in the liver and blood. These observations suggest that these probiotics can have an effect on liver steatosis.

Oral administration of P111, P121, or LpBL decreased HFD-induced proinflammatory cytokine expression, NF-κB activation (*p*-p65/065), and NF-κB-positive cell number in the colon, liver, and brain. Furthermore, they lowered HFD-induced LPS levels in the blood and feces. They also alleviated HFD-induced DCi-like behavior. LPS induces NF-κB-mediated proinflammatory cytokine expression, which triggers adipogenesis and hinders AMPK activation [4,5,6]. The AMPK activation inhibits systemic inflammation, including neuroinflammation [8,9]. Inflammation-inducing stressors such as pathogens and bacterial endotoxin induce DCi through systemic inflammation [10,11]. The combination of P111 with P121 (LpBl) additively alleviated HF-induced body weight gain, liver and EFP weights, liver steatosis, colitis, neuroinflammation, and DCi-like behavior. The efficacy of LpBl was more potent than those of P111 and P121, but not significantly. These findings suggest that P111 and/or P121 may suppress proinflammatory cytokine expression in the gut, liver, and brain by suppressing LPS-linked NF-κB signal, leading to the alleviation of colitis, hepatitis, neuroinflammation, and psychiatric disorders.

HFD feeding increased *Proteobacteria*, including *Helicobacteriaceae*, *Firmicutes*, *Streptococcaceae*, and *Staphylococcaceae* populations, and bacterial LPS production. However, LpBl, which most potently reduced HFD-induced body weight gain, suppressed HFD-induced populations of *Firmicutes* and *Proteobacteria*, in particular, *Streptococcaceae*, *Desulfovibrionaceae*, *Coriobacteriaceae*, and *Helicobacteriacae* populations, which had a positive correlation with body weight gain and a negative correlation with SIRT1 and BDNF expression levels in the liver. LpBl reduced the HFD-induced LPS level in the feces, blood, and liver that was positively correlated with TNF-α expression. The HFD-induced *Streptococcaceae* and *Helicobacteriaceae* number had a positive correlation with IT in the TST, while SA in the YMT were negatively correlated. However, LpBl treatment increased HFD-suppressed *Bacteroidetes* and *Verrucomicrobiota*, including *Akkermansiaceae*, *Prevotellaceae*, *Bifidobacteriaceae*, and *Enterobacteriaceae* populations, which had a negative correlation with DCi-like behavior and a positive correlation with an increased hippocampal BDNF expression level. Furthermore, LpBl suppressed HFD-induced corticosterone and IL-6 levels in the blood and IL-6 levels in the colon, blood, liver, and hippocampus. HFD feeding suppresses the *Akkermansiaceae* population in mice, in turn suppressing HFD-induced obesity and the blood glucose level [33,34,35]. The probiotic *L. plantarum* KY1032 increases *Akkermansiaceae* and *Bifidobacteriaceae* populations in volunteers with overweight [36]. Dietary fibers increase gut *Prevotellaceae* and *Bifidobacteriaceae* populations and reduce body weight gain. LPS increases DCi-like behavior and TNF-α and corticosterone expression [19]. *Lactobacillus reuteri* NK33 alleviates depressive symptoms in mice by inhibiting gut dysbiosis and bacterial LPS production. These results suggest that HFD can cause gut dysbiosis, which may be closely associated with body weight gain, liver steatosis, gut inflammation, neuroinflammation, and DCi-like behavior; and probiotics, in particular, LpBl, may mitigate obesity, liver steatosis, colitis, and DCi by alleviating gut dysbiosis and bacterial LPS production.

Moreover, we discovered that chronic feeding of an HFD could cause gut dysbiosis, along with body weight gain, which leads to DCi, and P111 and/or P121 could alleviate body weight gain and DCi by regulating gut dysbiosis and AMPK activation. However, to understand the action mechanism of P111 and/or P121, future research is needed to identify their substances and to clarify the pathogenesis of overgrown fecal bacteria stemming from an HFD.

## 5. Conclusions

P111, P121, and their mix LpBl can alleviate obesity, liver steatosis, DCi, and systemic inflammation, including colitis, liver inflammation, and neuroinflammation, in vivo by inducing AMPK activation and suppressing gut dysbiosis and LPS production.

## Data Availability

The datasets used and/or analyzed during the current study are available from the corresponding author upon reasonable request due to patient privacy.

## Supplementary Materials

The following supporting information can be downloaded at https://www.mdpi.com/article/10.3390/nu16223810/s1. Figure S1. Effects of P111 (Lpl) and P121 (Bio) on lipid accumulation and AMPK activation in 3T3 L1 and HepG2 cells. Figure S2. Characterization of the whole genomes of *L. plantarum* P111 and *Bifidobacterium longum* P21. Figure S3. Effects of P111 (Lpl) and P121 (Blo) on steatohepatitis-related marker expression in the liver. Figure S4. Effects of P111 (Lpl) and P121 (Bio) on neuroinflammation in the hippocampus of mice with HF-induced obesity. Figure S5. Effects of P111 and P121 on gut inflammation in the colon of mice with HF-induced obesity. Figure S6. Effect of LpBl on steatohepatitis-related marker expression in the liver. Figure S7. Effect of LpBl on neuroinflammation in the hippocampus of mice with HF-induced obesity. Figure S8. Effect of LpBl on gut inflammation in the colon of mice with HF-induced obesity. Table S1. Primers used in the present study. Table S2. Effect of LpBl on the fecal microbiota composition of HF-treated mice at the phylum level. Table S3. Effect of LpBl on the fecal microbiota composition of HF-treated mice at the family level. Methods—Behavioral tasks and determination of LPS in the blood, liver, and feces. Quantitative real-time polymerase chain reaction (qPCR). Gut microbiota composition analysis. Immunofluorescence staining. References [37,38,39,40] are cited in the Supplementary Materials.
