# Supplementary material for: Lactobacillus plantarum and Bifidobacterium longum Alleviate High-Fat Diet-Induced Obesity and Depression/Cognitive Impairment-like Behavior in Mice by Upregulating AMPK Activation and Downregulating Adipogenesis and Gut Dysbiosis
Source: Nutrients. 2024 Nov 7;16(22):3810. doi: 10.3390/nu16223810 (PMC11597813; doi:10.3390/nu16223810)
Supplement: Supplementary file 1 [file nutrients-16-03810-s001.zip › nutrients-3288673-supplementary.pdf]

[Supplement]

Lactobacillus plantarum and *Bifidobacterium longum* alleviate high-fat diet-induced obesity and depression/cognitive impairment-like behavior in mice by inducing AMPK activation and suppressing adipogenesis and gut dysbiosis

Table S1. Primers used in the present study

	Primer sequence	
	Forward	Reverse
SIRT1	5'-ATGACGCTGTGGCAGATTGTT-3'	5'-CCGCAAGGCGAGCATAGAT-3'
SREBP-1c	5'-AGCTGTCGGGGTAGCGTCTG-3'	5'-GAGAGTTGGCACCTGGGCTG-3'
PGC-1α	5'-CCGCCACCTTCAATCCAGAG-3'	5'-CAAGTTCTCGATTTCTCGACGG-3'
G6PD	5'-CCGGAAACTGGCTGTGCGCT-3'	5'-CCAGGTCACCCGATGCACCC-3'
FAS	5'-CTGAGATCCCAGCACTTCTTGA-3'	5'-GCCTCCGAAGCCAAATGAG-3'
LPL	5'-TGAAAGCCGGAGAGCTCAG-3'	5'-AGTGTCAGCCAGACTTCTTCAG-3'
Fiaf	5'-CACCCACTTACACAGGCCG-3'	5'-GAAGTCCACAGAGCCGTTCA-3'
β-actin	5'-TGTCCACCTTCCAGCAGATGT-3'	5'- AGCTCAGTAACAGTCCGCCTAGA -3'

Table S2. Effect of LpBl on the fecal microbiota composition of HF-treated mice at the phylum level.

Taxon Name	Mean±SD		
	LFD	HF	LpBl
Bacteroidetes	46.5±9.5	21.6±14.3	15.9±14.9
Firmicutes	41.3±7.3	58.2±10.3	64.4±17.3
Proteobacteria	7.7±4.7	18.8±11.7	16.2±4.3
Verrucomicrobia	3.6±4.8	0.0±0.0	2.1±4.3
Actinobacteria	0.2±0.1	0.4±0.2	0.4±0.4
Deferribacteres	0.1±0.3	0.5±0.6	0.4±1.1
Tenericutes	0.1±0.1	0.1±0.2	0.0±0.1
Cyanobacteria	0.1±0.1	0.0±0.0	0.0±0.1
Saccharibacteria_TM7	0.0±0.0	0.0±0.0	0.0±0.0

Table S3. Effect of LpBl on the fecal microbiota composition of HF-treated mice at the family level.

Taxon Name	Mean±SD		
	LFD	HF	LpBl
Muribaculaceae	31.5±8.2	10.2±5.4*	4.8±4.1[#]
Lachnospiraceae	18.1±7.4	22.0±6.0	28.5±10.0
Streptococcaceae	8.7±5.2	17.0±6.2*	12.9±8.2
Ruminococcaceae	8.2±1.4	15.2±5.2*	16.3±6.0
Rikenellaceae	6.8±5.1	4.1±3.8	4.7±6.0
Desulfovibrionaceae	5.4±3.2	14.6±11.5*	13.4±2.6
Bacteroidaceae	5.3±3.4	3.5±3.1	2.6±3.1
Akkermansiaceae	3.6±4.8	0.0±0.0	2.1±4.3
Helicobacteraceae	2.0±1.9	4.0±3.8*	2.5±3.2
Enterococcaceae	1.9±1.7	1.0±0.6	1.0±0.9
Lactobacillaceae	1.6±1.1	1.6±0.6	4.4±3.6[#]

Erysipelotrichaceae	1.4±2.4	0.1±0.1	0.2±0.2
Porphyromonadaceae	0.9±0.4	0.3±0.3*	0.2±0.1
AC160630_f	0.8±0.6	2.4±3.4	0.2±0.2
Christensenellaceae	0.7±0.6	0.3±0.1	0.2±0.1
Odoribacteraceae	0.5±0.5	0.9±0.6	1.3±1.6
Prevotellaceae	0.4±0.7	0.0±0.0	1.8±3.0
Dehalobacterium_f	0.2±0.0	0.2±0.0	0.2±0.0
Deferribacteraceae	0.1±0.3	0.5±0.6	0.4±1.1
Coriobacteriaceae	0.1±0.1	0.4±0.2*	0.4±0.4
FR888536_f	0.1±0.1	0.0±0.0	0.0±0.1
Enterobacteriaceae	0.1±0.1	0.0±0.0*	0.0±0.0
Peptococcaceae	0.0±0.0	0.2±0.1	0.0±0.0
Rhodospirillaceae	0.0±0.0	0.0±0.0	0.2±0.3
Clostridiaceae	0.0±0.1	0.1±0.1	0.0±0.1
Bifidobacteriaceae	0.0±0.0	0.0±0.0	0.0±0.0
Morganellaceae	0.0±0.0	0.0±0.0*	0.0±0.0
Mycoplasmataceae_f1	0.0±0.0	0.0±0.0	0.0±0.0

(a)

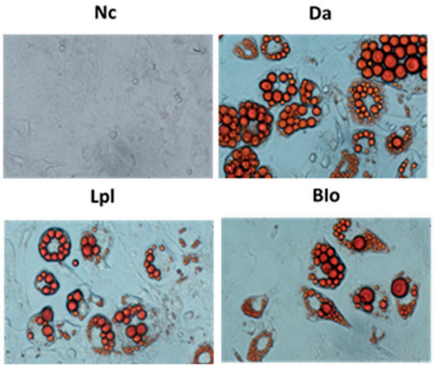

(b)

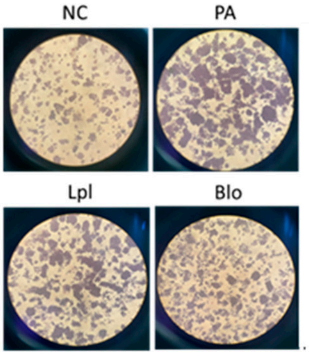

(c)

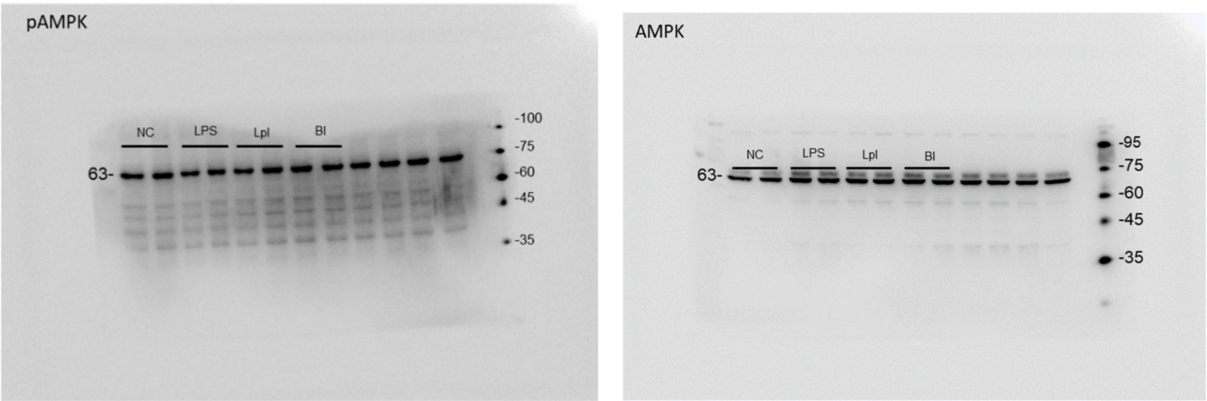

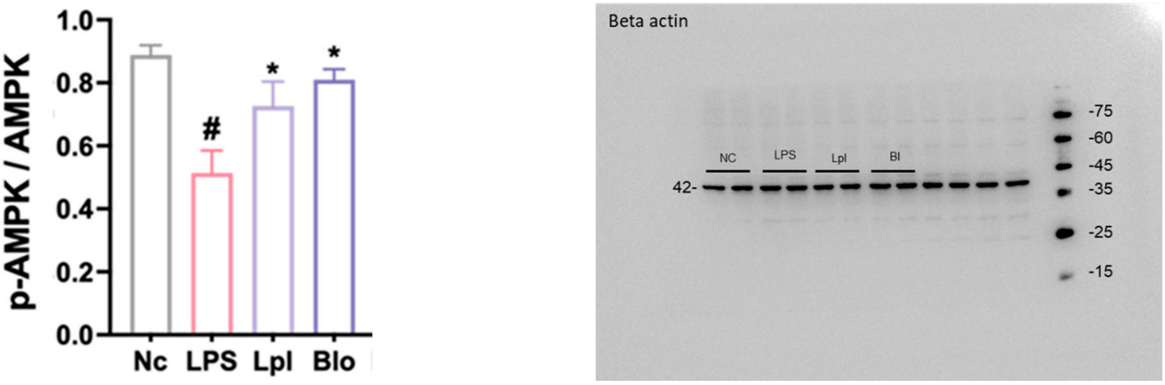

Figure S1. Effects of P111 (Lpl) and P121 (Bio) on lipid accumulation and AMPK activation in 3T3 L1 and HepG2 cells. (a) Effects on lipid accumulation in dexamethasone (Da)-stimulated 3T3 L1 cells, assessed by Oil red O staining. (b) Effects on lipid accumulation in palmitic acid (PA)-treated HepG2 cells, assessed by Oil red O staining. (c) Effects on AMPK activation in HepG2 cells, assessed by immunoblotting, respectively and immunoblotting, respectively. 3T3 L1 cells or were treated with probiotics (1×10^5 CFU/mL) in the presence of dexamethasone. HepG2 cells were treated with probiotics (1×10^5 CFU/mL) with palmitic acid. Data are presented as mean ± S.D. (n=4). $^{\#}p < 0.05$ vs. LF group. $^{*}p<0.05$ vs. HF group.

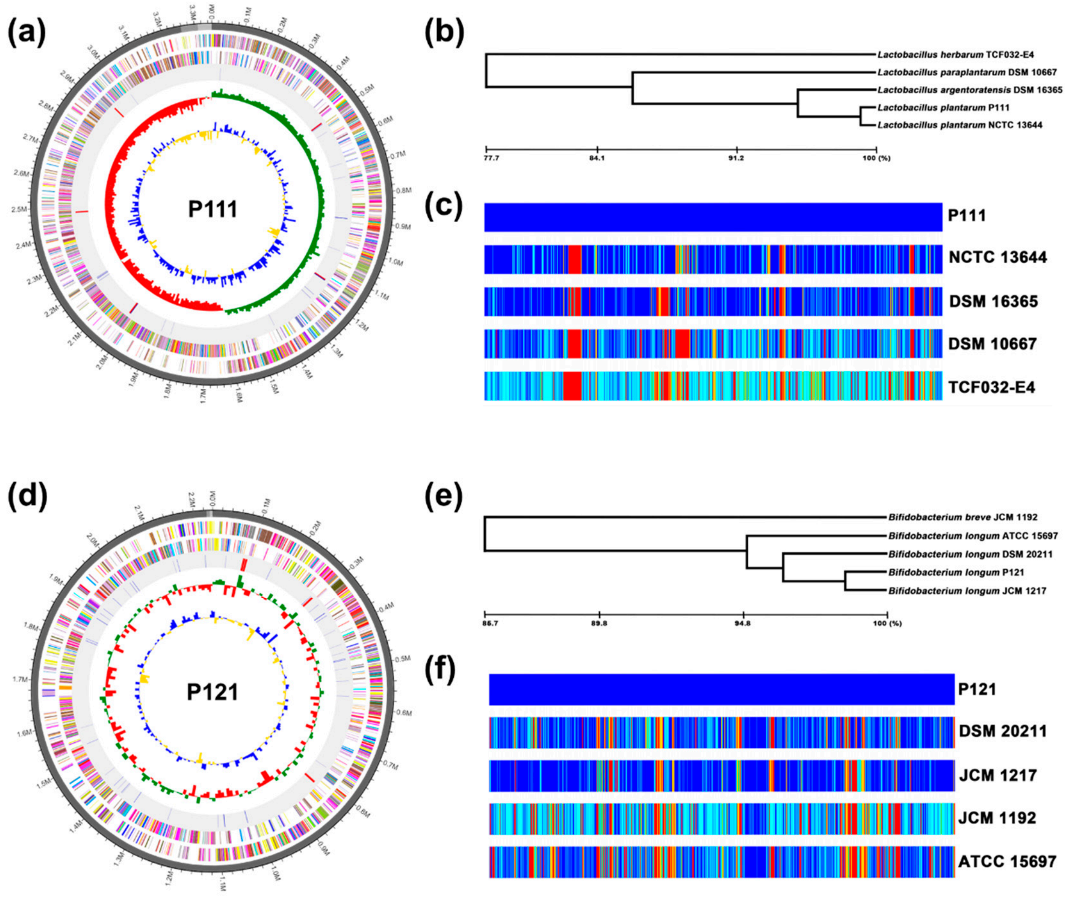

Figure S2. Characterization of the whole genomes of L. plantarum P111 and Bifidobacterium longum P21. (a) P111 pseudochromosome drawn from 3 contig: outermost circle, contig; inner circle, the color coded for the CDS information analyzed in the forward strand; and third circle,

the CDS information analyzed in the reverse strand; fourth circle, tRNA (blue) and rRNA (red). (b) The pairwise ortholog matrix table of P111. (c) P111 neighbor-joining tree based on the OrthoANI distance matrix. (d) P121 Bpseudochromosome drawn from 3 contig. (e) The pairwise ortholog matrix table of P121. (f) P121 neighbor-joining tree based on the OrthoANI distance matrix.

(a)

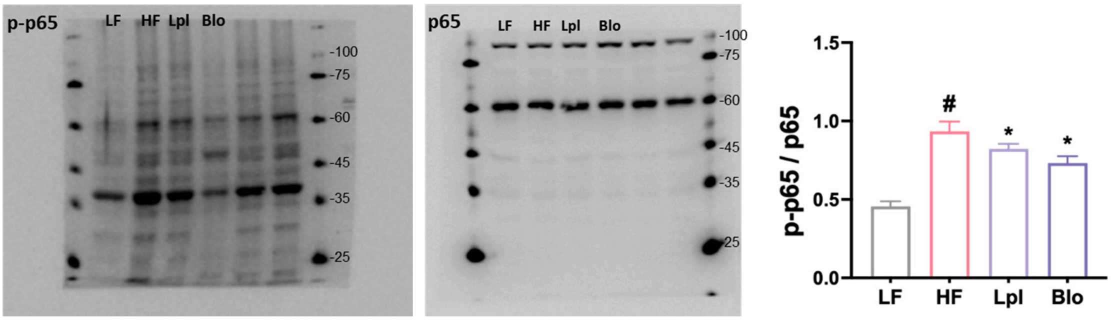

(b)

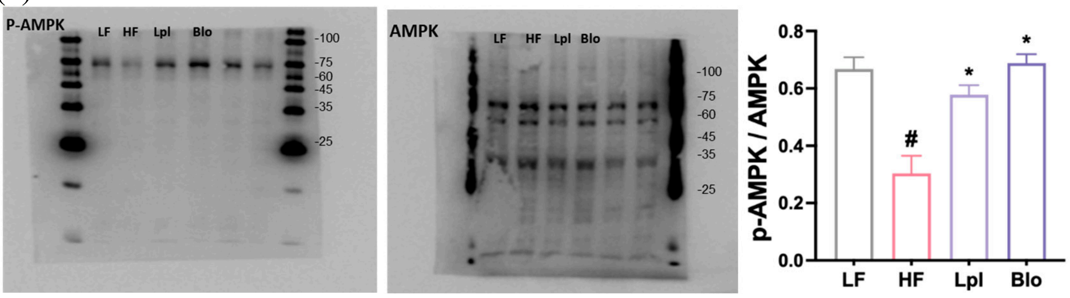

(c)

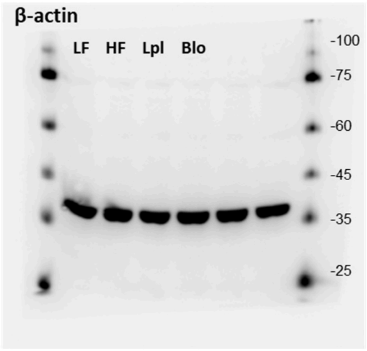

Figure S3. Effects of P111 (Lpl) and P121 (Blo) on steatohepatitis-related marker expression in the liver. Effects on p-p65 and p65 expression (a), p-AMPK and AMPK expression (b), and β-actin expression (c), assessed by immunoblotting. LF, low-fat diet (8 weeks) alone; HF, high-fat diet (8 weeks) alone; Lpl, P111 (4 weeks) with high-fat diet (8 weeks); Blo, P121 (4 weeks) with high-fat diet (8 weeks). Data are presented as mean ± S.D. (n=8). $^{\#}p < 0.05$ vs. LF group. $*p<0.05$ vs. HF group.

(a)

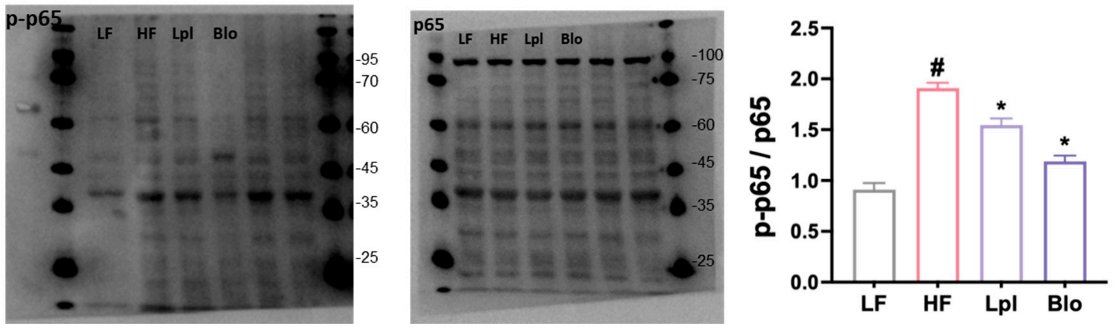

(b)

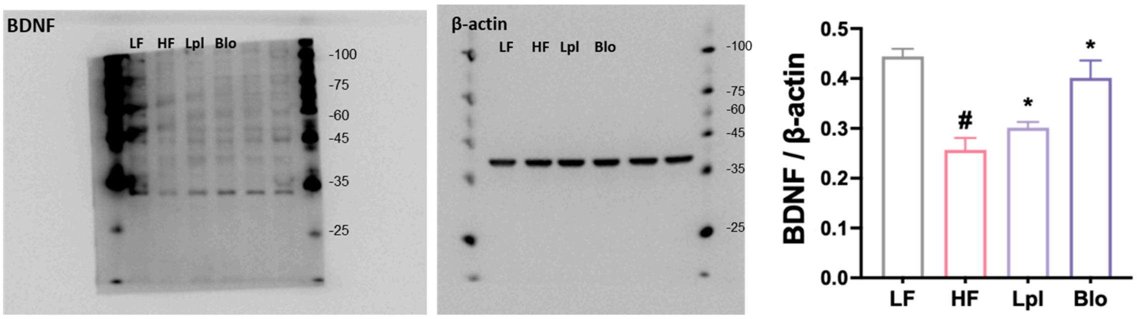

Figure S4. Effects of P111 (Lpl) and P121 (Bio) on neuroinflammation in the hippocampus of mice with HF-induced obesity. Effects on hippocampal p-p65 and p65 expression (a), BDNF (b), and β-actin expression (c), assessed by immunoblotting. LF, low-fat diet (8 weeks) alone; HF, high-fat diet (8 weeks) alone; Lpl, P111 (4 weeks) with high-fat diet (8 weeks); Blo, P121 (4 weeks) with high-fat diet (8 weeks). Data are presented as mean ± SD (n=8). $^{\#}p < 0.05$ vs. LF group. $^{*}p<0.05$ vs. HF group.

(a)

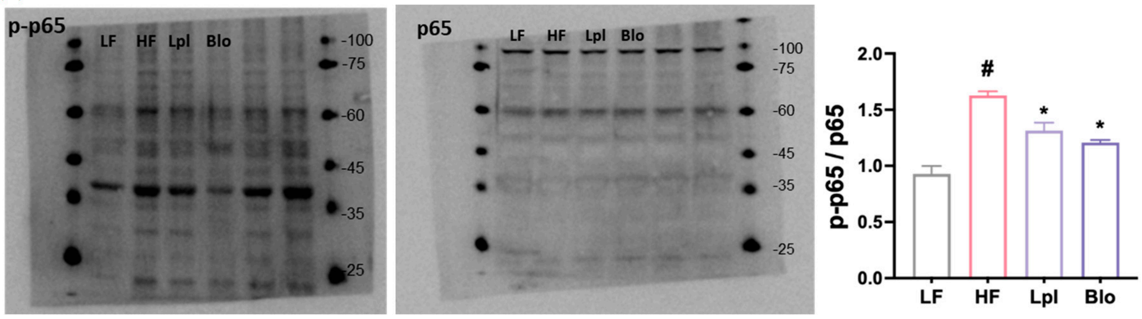

(b)

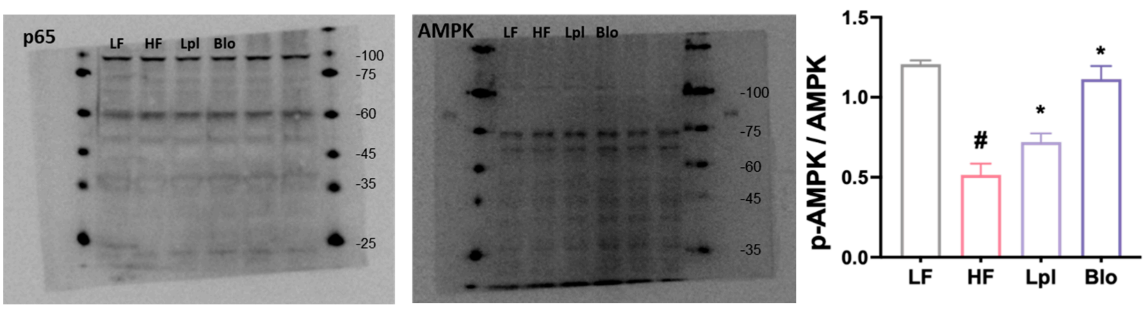

(c)

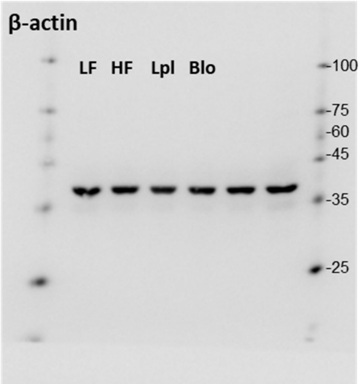

Figure S5. Effects of P111 and P121 on gut inflammation in the colon of mice with HF-induced obesity. Effects on colonic p-p65 and p65 expression (a), p-AMPK and AMPK expression (b), and β-actin expression (c), assessed by immunoblotting. LF, low-fat diet (8 weeks) alone; HF, high-fat diet (8 weeks) alone; Lpl, P111 (4 weeks) with high-fat diet (8 weeks); Blo, P121 (4 weeks) with high-fat diet (8 weeks). Data are presented as mean ± S.D. (n=8). $^{\#}p < 0.05$ vs. LF group. $^{*}p<0.05$ vs. HF group.

(a)

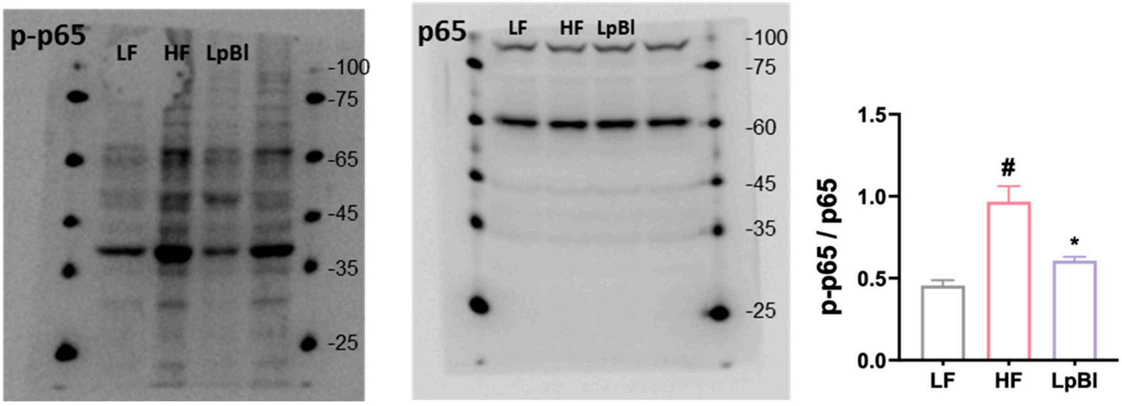

(b)

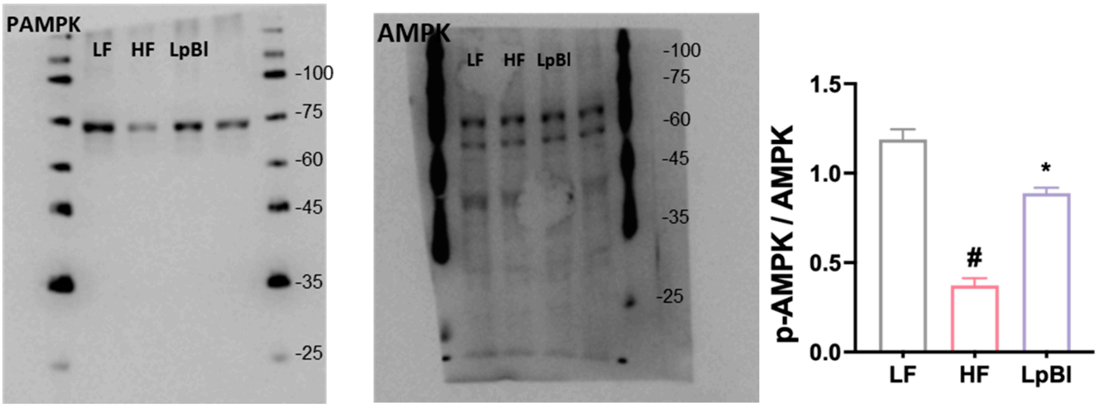

(c)

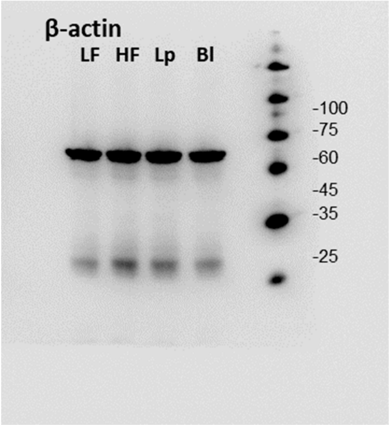

Figure S6. Effect of LpBl on steatohepatitis-related marker expression in the liver. Effects on p-p65 and p65 expression (a), p-AMPK and AMPK expression (b), and β-actin expression (c). LF, low-fat diet (8 weeks) alone; HF, high-fat diet (8 weeks) alone; LpBl, P111 and P121 mix (4 weeks) with high-fat diet (8 weeks).

(a)

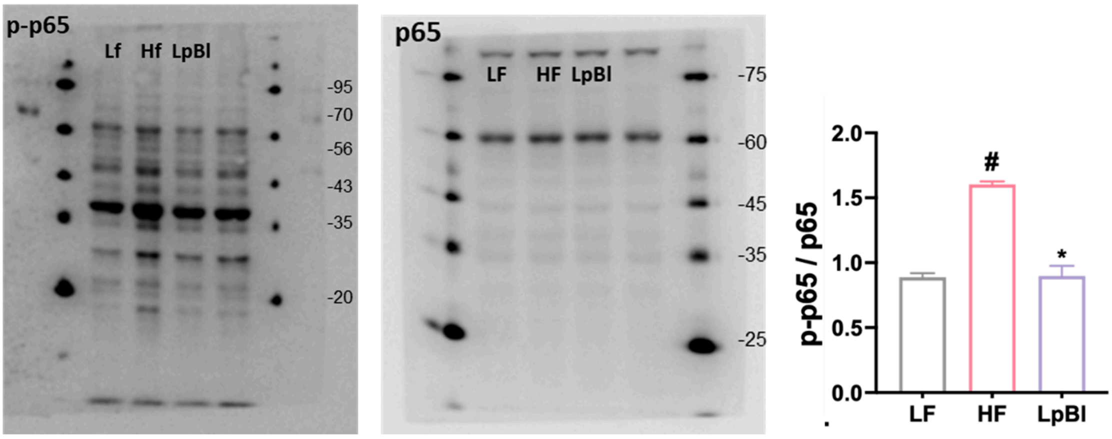

(b)

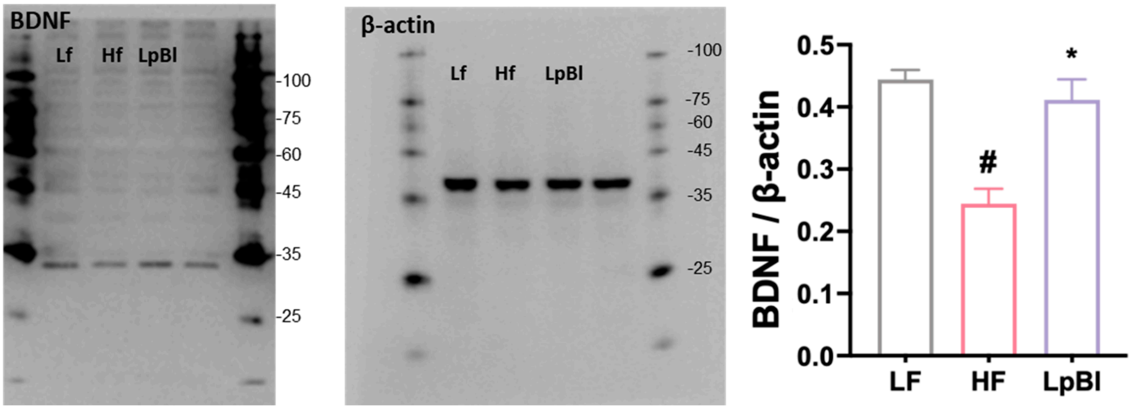

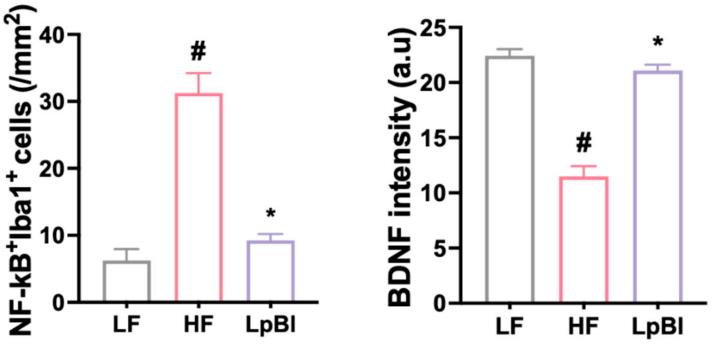

Figure S7. Effect of LpBl on neuroinflammation in the hippocampus of mice with HF-induced obesity. Effects on hippocampal p-p65 and p65 expression (a) and BDNF and β-actin expression (b), assessed by immunoblotting. Effects on the intensity of hippocampal NF-κB^{+}Iba1^{+} (c) and BDNF^{+}NeuN^{+} cell populations (d), assessed by confocal microscopy. LF, low-fat diet (8 weeks) alone; HF, high-fat diet (8 weeks) alone; Lpl, P111 (4 weeks) with high-fat diet (8 weeks); Blo, P121 (4 weeks) with high-fat diet (8 weeks). Data are presented as mean ± S.D. (n=8). $^{\#}p < 0.05$ vs. LF group. $^{*}p<0.05$ vs. HF group.

(a)

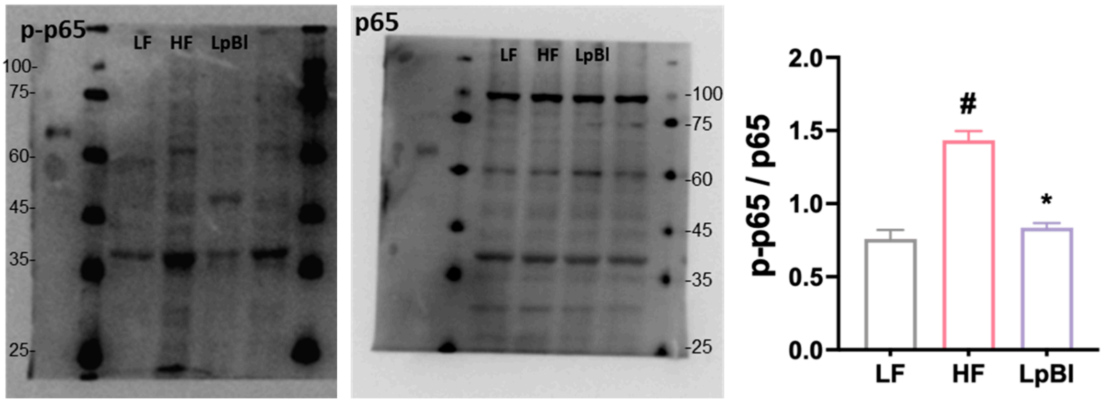

(b)

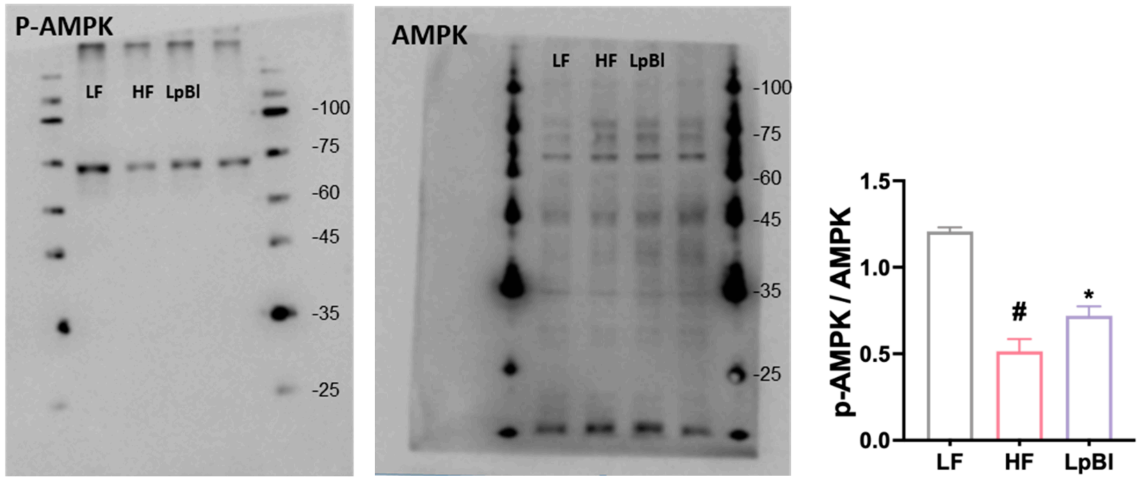

(c)

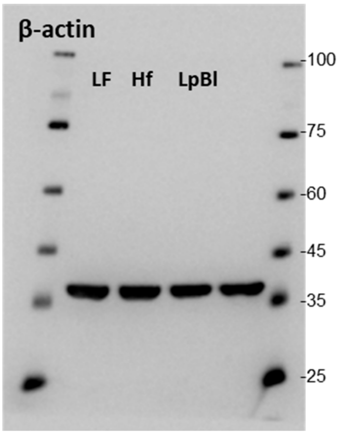

Figure S8. Effect of LpBl on gut inflammation in the colon of mice with HF-induced obesity. Effects on colonic p-p65 and p65 expression (a), p-AMPK and AMPK expression (b), and β-actin expression (c), assessed by immunoblotting. LF, low-fat diet (8 weeks) alone; HF, high-fat diet (8 weeks) alone; LpBl, P111 and P121 mix (4 weeks) with high-fat diet (8 weeks). Data are presented as mean ± S.D. (n=8). $^{\#}p < 0.05$ vs. LF group. $^{*}p<0.05$ vs. HF group.

Methods

Behavioral tasks

The Y-maze task was carried out according to the method of Lee et al. [1]. Each mouse was initially placed in the center of the Y-maze, and within one arm, a three-arm horizontal maze (40 [length] × 3 [width] × 12 cm [height]) and the sequence of arm entries were recorded for 8 min. A spontaneous alternation was defined as an entry into all three arms consecutively. The spontaneous alteration (%) was indicated as the ratio of the spontaneous to possible alternations.

The elevated plus maze (EPM) task was performed in the plus-maze apparatus according to the method of Jang et al. [2]. The maze consisted of two open (30 × 7 × 1 cm) and two enclosed arms (30 × 7 × 20 cm, each) extending from a central platform (7 × 7 cm) elevated to a height of 50 cm above the floor. Time spent in open arms and open arm entries of each mouse were measured in video camera-installed dimly lit room (20 lux) for 5 min. The maze was cleaned with 70% ethanol to remove residues and odours before every trial. The percentage of time spent in open arms [(time spent in open arms/time spent in open and closed arms) ×100] and of open arm entries [(the number of open arm entries/the number of entries of open and closed arms) ×100] were calculated.

A tail suspension test (TST) was measured according to the method of Jang et al. Mice were suspended on the edge of a table 30 cm above the floor by taping 1 cm from the tail tip. Immobility time was measured for 5 min. When the mice did not move and passively hung, mice were judged to be immobile.

Determination of LPS in the blood, liver, and feces

The contents of LPS in the blood, liver, and feces were assayed by using the diazo-coupled limulus amoebocyte lysate (LAL) assays according to the method of Kim et al. [3]. For the LPS assay in the blood, bloods collected by retroorbital bleeding into ethylenediaminetetraacetic acid-coated BD Microtainer® tubes (Becton Dickinson, Franklin Lakes, NJ, USA) were centrifuged at 13,200 *g* for 15 min. The supernatant (5 μL) was diluted 1:10 in pyrogen-free water and inactivated for 10 min at 70°C. For the fecal and liver LPS assay, feces were placed in 50 mL of PBS, sonicated for 1 h on ice, and centrifuged at 400 *g*

for 10 min. Liver were homogenized with RIPA lysis buffer and centrifuged at 400 *g* for 10 min. Supernatants were filtrated through a 0.45 μm Millipore filter, re-filtrated through a 0.22 μm filter, and inactivated at 70°C for 10 min. LPS contents of filterates and supernatants (50 μl) were assayed using LAL assay kit according to the manufacturer's protocol.

Quantitative real time-polymerase chain reaction (qPCR)
qPCR for SIRT-1, SREBP-1c, PGC-1α, G6PD, FAS, LPL, Fiaf, and β-actin was performed with 2 μg of total RNA isolated from the liver, by utilizing Takara thermal cycler, which used SYBER premix agents according to the method of Jang et al. [4]. Thermal cycling conditions were as follows: activation of DNA polymerase at 95℃ for 30 s, followed by 40 cycles of amplification at 95℃ for 15 s and at 60℃ for 30 s. The normalized expression of the assayed genes, with respect to β-actin, was computed for all samples by using the Microsoft Excel data spreadsheet.

Gut microbiota composition analysis
Pyrosequencing of gut microbiota was performed according to the method of Kim et al. [5]. Genomic DNAs of gut microbiota were isolated from the fresh feces of mice using a QIAamp Fast DNA stool mini kit. PCR amplification was carried out by using primers targeting the V3 to V4 regions of 16S ribosomal RNA genes with gut bacterial genomic DNA. For the amplification, barcode-containing fusion primers were used. The sequencing for equimolar concentration of each amplicon was carried out at Chunlab Inc. (Seoul, Korea) with Illumina MiSeq System, as stated in the manufacturer's directions. Reads taken from different samples were classified by unique barcodes of each PCR product and the target region in barcoded primers was identified. All of the linked sequences including adapter, barcode, and linker and low quality sequences (reads with two or more indefinite nucleotides, a low quality score, or < 500 bp) were eliminated. Potential chimeric sequences were confirmed by the Bellerophon formula. The taxonomic sorting of each read was assigned against the EzTaxon-e database (http://eztaxon-e.ezbiocloud.net), which has the 16S rRNA gene sequence of type strains that have valid published names and representative species level phylotypes of either cultured or uncultured entries in the GenBank database with complete hierarchical taxonomic classification from phyla to species. The species richness of samples was determined using the CLcommunity program. Subsampling was randomly performed to equalize the read size of tested samples to compare the different read size within tested samples. For the comparison of the OTUs between tested samples, shared OTUs were obtained with the XOR analysis of the CLcommunity program.

Immunofluorscence staining
Their brain, vagina, tissues of mice transcardially perfused with formaldehyde were collected, post-fixed, cytoprotected, frozen, and sectioned. The sections were incubated with primary antibodies against brain-derived neurotropic factor (BDNF), neuronal nuclei antigen (NeuN), NF-κB, Iba1, CD11c, TNF-α, and/or RANK for 12 h, treated with secondary antibodies conjugated with Alexa Fluor 594 or Alexa Fluor 488 (1:200, Invitrogen) for 2 h, then stained with 4′,6-diamidino-2-phenylindole, dilactate (DAPI), and observed using a confocal laser microscope.

References
1. Lee, H.J.; Lee, K.E.; Kim, J.K.; Kim, D.H. Suppression of gut dysbiosis by Bifidobacterium longum alleviates cognitive decline in 5XFAD transgenic and aged mice. *Sci Rep* **2019**, *9*, 11814, doi:10.1038/s41598-019-48342-7.

2. Jang, H.M.; Lee, K.E.; Kim, D.H. The Preventive and Curative Effects of Lactobacillus reuteri NK33 and Bifidobacterium adolescentis NK98 on Immobilization Stress-Induced Anxiety/Depression and Colitis in Mice. *Nutrients* **2019**, *11*, doi:10.3390/nu11040819.
3. Kim, K.A.; Gu, W.; Lee, I.A.; Joh, E.H.; Kim, D.H. High fat diet-induced gut microbiota exacerbates inflammation and obesity in mice via the TLR4 signaling pathway. *PLoS One* **2012**, *7*, e47713, doi:10.1371/journal.pone.0047713.
4. Jang, H.M.; Han, S.K.; Kim, J.K.; Oh, S.J.; Jang, H.B.; Kim, D.H. Lactobacillus sakei Alleviates High-Fat-Diet-Induced Obesity and Anxiety in Mice by Inducing AMPK Activation and SIRT1 Expression and Inhibiting Gut Microbiota-Mediated NF-κB Activation. *Mol Nutr Food Res* **2019**, *63*, e1800978, doi:10.1002/mnfr.201800978.
5. Kim, J.K.; Lee, K.E.; Lee, S.A.; Jang, H.M.; Kim, D.H. Interplay Between Human Gut Bacteria Escherichia coli and Lactobacillus mucosae in the Occurrence of Neuropsychiatric Disorders in Mice. *Front Immunol* **2020**, *11*, 273, doi:10.3389/fimmu.2020.00273.
